# Neurophysiological biomarkers of treatment response in suicidal ideation: a systematic review

**DOI:** 10.1038/s41398-025-03477-2

**Published:** 2025-11-17

**Authors:** Noah Stapper, Lindsay L. Benster, Sahit Menon, Emma C. Boyd, Mohsen Poorganji, Itay Hadas, Yinming Sun, Lawrence G. Appelbaum, Zafiris J. Daskalakis, Cory R. Weissman

**Affiliations:** 1 SDSU UC San Diego Joint Doctoral Program in Clinical Psychology, San Diego, CA USA; 2 Department of Psychiatry, University of California, La Jolla, San Diego, CA USA

**Keywords:** Predictive markers, Psychiatric disorders

## Abstract

**Background:**

Suicidal ideation (SI) is associated with increased morbidity and is one of the main modifiable risk factors for suicide. While initial evidence indicates the efficacy of several treatments for SI, most treatments were not developed to specifically target SI and are often associated with side effects or high relapse rates. Limited understanding of the neurophysiological basis of SI has hindered the optimization of these treatments.

**Methods:**

This systematic review synthesizes the evidence on neurophysiological biomarkers associated with treatment-induced changes in SI in the context of clinical trials. A systematic literature of the Embase, PubMed, and PsycInfo databases was conducted according to the Preferred Reporting Items for Systematic Review and Meta-Analyses (PRISMA) guidelines.

**Results:**

Twenty-four articles were eligible for inclusion in this review, with most published within the past five years. The studies showed methodological heterogeneity, leading to limited convergence in findings. Many studies were limited by non-randomized study design, concurrent interventions, incomplete treatment protocols, and unvalidated assessments of SI. Despite these limitations, the findings suggest the involvement of the anterior cingulate cortex (ACC) in the anti-suicidal effects of intravenous (IV) ketamine. Notably, this effect was absent in patients treated with oral ketamine, possibly explaining the clinically superior anti-suicidal effects of IV-ketamine compared to the oral administration. Improvements in SI following electroconvulsive therapy and magnetic seizure therapy were associated with activity in the prefrontal cortex (PFC).

**Conclusion:**

These findings may indicate that the differential modulation of the ACC and PFC is linked to the acute, yet transient effects of IV-ketamine and the sustained effects of seizure therapies. Future studies designed to prospectively assess the efficacy of SI treatments should include these potential biomarkers of treatment response in their design.

## Background

Suicide is a major societal and public health concern [1], accounting for approximately 700,000 deaths annually worldwide [2]. In the US, suicide is the second leading cause of death for people between the ages of 20 and 34 [3]. Suicidal ideation (SI), which includes thoughts, considerations, and plans related to suicide [4], is associated with significant morbidity [5, 6] and represents a modifiable risk factor for suicide deaths. Individuals endorsing SI experience a three times higher risk of suicide compared to those that do not report SI [7, 8], and 29% of people reporting SI attempt suicide [6, 9]. In research, SI is commonly employed as a proxy for suicide due to its higher lifetime prevalence, estimated at around 10–18% [10], rendering it as a focal point for suicide prevention efforts [11].

Several neuromodulatory, psychotherapeutic, and pharmacological treatment strategies demonstrate efficacy in reducing SI severity. Electroconvulsive therapy (ECT) is the most robust anti-suicidal treatment, with a response rate of 60–80% [12, 13]. Nonetheless, utilization of ECT is constrained by limited access, negative stigma and cognitive side effects [14]. Intravenous (IV) ketamine demonstrates acute anti-suicidal effects, however there is limited evidence of sustained response [15, 16]. Repetitive transcranial magnetic stimulation (rTMS) treatment leads to remission of SI in 20–40% of patients, depending on the protocol [17, 18]. Psychotherapeutic interventions, such as dialectical behavior therapy (DBT) or cognitive behavioral therapy (CBT), have shown mild to moderate effect sizes in reducing SI [16, 19], however, the treatment response for psychotherapy often takes several months [20] and, therefore are less effective in reducing acute SI. Results on the efficacy of pharmacological interventions for SI are heterogeneous. Lithium and clozapine are effective at reducing SI in patients primarily with bipolar disorder and schizophrenia, respectively [21]. Several serotonin reuptake inhibitors (SSRIs) and serotonin-noradrenaline reuptake inhibitors (SNRI’s) demonstrate robust evidence for the treatment of Major Depressive Disorder (MDD) [22–24], which is relevant given that a large proportion (40.3%) [25] of patients diagnosed with MDD report SI in their lifetime. However, two studies have found that 8.6% [26] and 14.3% [27] of patients with major depressive disorder (MDD) taking SSRIs experience an increase in SI following treatment. These adverse effects of SSRIs are especially common among young adults and present a major limitation of these pharmacological treatments in this population [28].

Although the treatments described above have been shown to reduce SI, most patients endorsing SI do not respond to treatment [13, 18, 19]. These treatments were primarily developed to target neurophysiological features of other psychiatric conditions, such as MDD and schizophrenia; consequently, they may only indirectly modulate symptoms of SI [29]. For this reason, understanding the neurophysiology of SI is critical for developing interventions that specifically target SI symptoms, thereby enhancing response rates and preventing future suicide deaths. One previous narrative review by Schmaal and colleagues [30] summarized the findings of cross-sectional studies exploring the neurobiological basis of SI and suicidal behavior. They found that the prefrontal cortex (PFC), cingulate, insula, hippocampus, amygdala, thalamus and striatum regions were involved in SI and suicidal behavior. The authors hypothesized that this involvement may be attributed to disturbances of the roles of these areas in impulse control and emotion regulation. Although the number of studies specifically investigating the neurobiology of SI is limited, they found that patients endorsing SI, compared to diagnosis matched controls, exhibited altered activity in the cingulate, orbitofrontal cortex (OFC) and temporal gyrus [30]. While cross-sectional studies are important for the characterization of the underlying neurobiology of SI, the results of this study offer limited clinical utility for informing causal and modifiable targets for optimized neuromodulatory treatments for SI. Therefore, this systematic review synthesizes prior studies investigating the neurophysiological correlates of changes in SI following treatment. We hypothesized that patterns of biomarkers correlated with changes in SI would emerge across imaging modalities and treatments. However, given the limited number of studies and small sample sizes in this field, along with heterogeneity in treatment protocols, some treatments may not demonstrate a replicable SI biomarker. The results of this study will provide insight into the neurophysiological mechanisms of treatments for SI and may inform the development of optimized interventions that directly target the symptom construct of SI.

## Methods

**Fig. 1:**
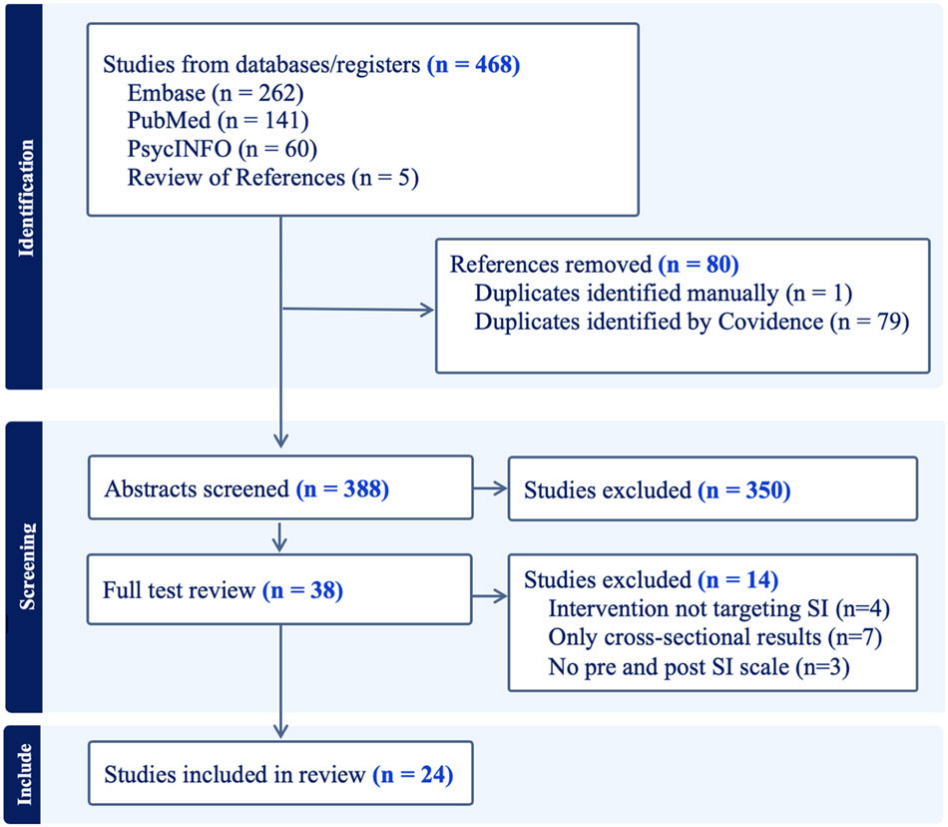
PRISMA diagram.

We conducted a literature search according to the Preferred Reporting Items for Systematic Review and Meta-Analyses (PRISMA) guidelines. The study was prospectively registered on PROSPERO with the identification number: CRD42023472176. The literature search of electronic databases was conducted using key words and standard vocabulary in Embase, PubMed, and PsycInfo databases on October 23rd, 2023, with an updated PubMed search on February 21st, 2024 (see Supplemental Materials for search statement). Additional articles were identified through forward and backward bibliometric searches of relevant articles. Two investigators (NS and LB) manually screened the titles and abstracts of papers identified through the database search and subsequently conducted full text reviews of eligible papers (Fig. 1). The following information was extracted from the eligible articles: title, year, country of data collection, functional neuroimaging and neurophysiological method (type, regions of interest, pre-processing software), treatment protocol (type, number of sessions, timeframe of sessions, dose, target region), sample size, population (diagnosis, baseline SI severity, level of treatment resistance, concurrent medication, age), SI scale used, study design, and main outcomes (e.g. variables, *p* value, correlation value, statistical test used).

Peer-reviewed studies were included based on the following criteria: 1) Participants reported SI or a suicide attempt (defined as self-directed, non-fatal and potentially injurious behavior with the intent to die [4]), 2) received a treatment, 3) and underwent functional neuroimaging. Studies were eligible if subjects reported SI at the time of treatment or a suicide attempt in the preceding six months. The use of a validated scale or subscale of SI or suicide attempts was required and there were no restrictions regarding the subjects’ age or psychiatric diagnosis. Treatment interventions considered for inclusion fell within three categories: neuromodulation (e.g. ECT or TMS), psychotherapy (e.g. CBT), or psychopharmacology (e.g. ketamine, or antidepressants). Functional neuroimaging and neurophysiological techniques include electroencephalogram (EEG), intracranial EEG (iEEG), functional magnetic resonance imaging (fMRI), TMS combined with EEG (TMS-EEG), TMS-electromyography (TMS-EMG), TMS-fMRI, positron emission tomography (PET), single-photon emission computed tomography (SPECT), functional near-infrared spectroscopy (fNIRS), and magnetoencephalography (MEG). Neurophysiological recordings were required either pre-treatment, to identify neurophysiological predictors of treatment response, or pre- and post-treatment to document treatment-related changes. Additionally, studies were required to include a statistical analysis of the relationship between the change in suicidality and neurophysiological markers. Reviews, meta-analyses, opinion pieces, case reports or conference abstracts were excluded.

## Results

**Fig. 2:**
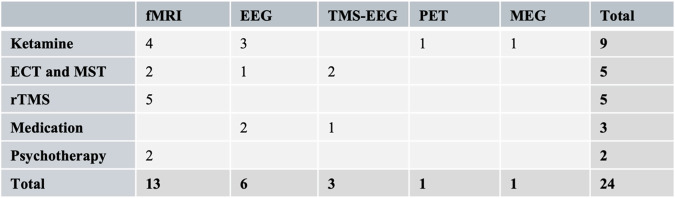
Summary of eligible studies.

The literature search yielded 388 articles; 38 articles underwent full text review, and 24 studies ultimately met eligibility criteria to be included in this review (Fig. 1). Most articles (18) were published within the last six years, including six articles in 2023. Most studies were conducted either in China (eight) or the USA (six), and many of the studies involved resting-state fMRI (13) or resting-state EEG (six). Eight of the studies were randomized controlled trials (RCT) and 15 studies were open label. Nine studies involved the administration of Ketamine (six intravenous, three oral), five utilized TMS, five with ECT or MST, three involved a pharmacological intervention, and two with psychotherapy (Fig. 2).

Most included studies (22) enrolled patients with a diagnosis of MDD. Among these, nine studies required a diagnosis of treatment-resistant depression (TRD). Definitions of TRD varied, but most studies classified TRD as the lack of treatment response to at least two antidepressants at an adequate dose and duration. Two studies enrolled patients that had a diagnosis of either bipolar disorder [31] or post-traumatic stress disorder (PTSD) [32]. The two remaining studies included patients reporting chronic SI (SSI > 6), irrespective of diagnosis [33, 34]. The mean age of patients across the included studies was 36.9 (+/−10.47) years. Two studies exclusively enrolled adolescent patients [35, 36].

To assess symptoms of SI, 17 studies used a full validated scale measuring SI, of which 14 used the Beck Scale for Suicidal Ideation (BSSI) [37], and seven used a single sub-item from a depression scale to quantify SI severity [32, 38–43]. Baseline SI severity scores demonstrated notable heterogeneity, ranging from mild to severe SI. Average baseline BSSI scores ranged from 5.53 [31] to 21.43 [36].

### Seizure therapies (ECT/MST)

ECT is a non-invasive neuromodulation technique that induces a short, generalized seizure under general anesthesia. The procedure involves the application of an electrical current via two electrodes placed on the scalp over the frontal cortex [44]. Magnetic seizure therapy (MST) is another non-invasive seizure therapy. In MST seizures are induced by eddy currents generated via electromagnetic induction [45]. The electrical current induced by MST is more focal than that of ECT and produces less cognitive side effects [46]. While ECT has the most robust evidence as a treatment for SI [13], there remains a scarcity of research examining the neurophysiological correlates of an improvement in SI following ECT treatment. In this review we identified two studies [36, 47] exploring the effects of ECT, and three studies [48–50] that investigated the neurophysiological effects of MST (Table 1). A variety of neurophysiological approaches were used: two studies utilized fMRI, and others used TMS-EEG and resting state EEG. Notably, all five studies identified a neural biomarker within the frontal cortex, which was significantly associated with an improvement in SI following treatment. Specifically, four studies found a neural biomarker of SI treatment response in the prefrontal cortex and one study in the right precentral gyrus.

**Table 1:** Neurophysiological biomarkers of SI improvement following ECT or MST treatment.

ECT & MST	Imaging Method	Treatment protocol (1. Type 2. Sessions, 3. Timeframe 4. Dose, 5. Target region)	Sample size	Population (1. Diagnosis, 2. Baseline SI, 3. concomitant medication, 4. Age)	SI Response (1. Scale, 2. Pre SI 3. Post SI, 4. *P*-value)	Design	Main Result
Wang, [47]	fMRI	1. ECT 2. 8–12 sessions 3. Up to 3 weeks 4. Half age method 5. Bifrontal	26	1. MDD 2. 43.74 ± 20.83 (R), 68.69 ± 22.95 (NR) 3. All patients took antidepressant medications, 5 patients had a change of antidepressants 4. 27.73 ± 7.59 (16–45)	1. BSSI 2. 43.74 ± 20.83 (R), 68.69 ± 22.95 (NR) 3. 0.00 (R), 45.45 ± 12.12 (NR) 4. *p* < 0.001 (R), *p* = 0.086 (NR)	Open label	Increases in fALFF in the orbitofrontal cortex and left superior frontal gyrus are significantly associated with a reduction in SI following ECT
Li, [36]	fMRI	1. ECT 2. 10 3. Three times daily on consecutive days, then every second day for two weeks 4. Half age method	14	1. MDD and chronic SI inpatients 2. 21.43 (3.67) 3. Patients were prescribed a new medication at onset of study 4. 14.57 (1.45)	1. BSSI 2. 21.43 ± 3.67 3. 6.93 ± 3.58 4. *p* < 0.001	Open label	A decrease in SI following ECT treatment is significantly associated with an increase in fALFF in the right precentral gyrus
Sun, [50]	TMS-EEG	1. MST 2. 24 sessions or until remission 3. Not reported 4. Mean seizure duration 45.1 (±21.4) 5. Over the Fz electrode	23	1. TRD 2. 9.3 ± 6.3 3. Concurrent medication kept stable prior to study 4. 45.0 ± 12.2	1. BSSI 2. 9.3 ± 6.3 3. 4.3 ± 5.6 4. *p* < 0.001	Open label	The reduction in SI symptoms following MST treatment is significantly correlated with a reduction in LICI in the DLPFC.
Sun, [49]	TMS-EEG	1. MST 2. 24 sessions or until remission 3. Not reported 4. Mean seizure duration 45.1 (±21.4) 5. Over the Fz electrode	27	1. TRD 2. 9 ± 6.8 3. Concurrent medication kept stable prior to study 4. 46.0 ± 15.3	1. BSSI 2. 9.0 ± 6.8 3. 4.2 ± 6.3 4. *p* = 0.001	Open label	Greater improvement in SI following MST is associated with a more negative N100 amplitude at baseline and greater baseline LICI values over the frontal electrode.
Atluri, [48]	EEG	1. MST 2. Until remission (max 24 sessions) 3. 2–3 times weekly 4. 25–100 hz MST 5. DMPFC	24	1. TRD 2. All patients endorsed SI at baseline. 3. Psychotropic medications were not discontinued prior to study 4. 45.7 ± 14.4	1. BSSI 2. Not reported 3. Not reported 4. *p* = 0.0002	Open label	Baseline EEG microstates significantly predict improvement in SI following MST treatment

*ECT* electroconvulsive therapy, *MST* magnetic seizure therapy, *fMRI* functional magnetic resonance imaging, *EEG* electroencephalogram, *TMS-EEG* concurrent TMS and EEG, *DMPFC* dorsomedial prefrontal cortex, *MDD* major depressive disorder, *R* responder, *NR* non-responder, *TRD* treatment resistant depression, *BSSI* beck scale for suicidal ideation, *fALFF* fractional amplitude of low-frequency fluctuations, *DLPFC* dorsolateral prefrontal cortex, *LICI* long interval cortical inhibition.

Two open-label resting state fMRI studies [36, 47] investigated the neurophysiological effects of ECT for SI using fractional amplitude of low-frequency fluctuations (fALFF). This fMRI metric captures the intensity of spontaneous neuronal activity within localized brain regions. fALFF is calculated by comparing the BOLD signal at specific low frequencies to the total amplitude across all frequency bands. The findings suggest an association between SI treatment response and increased fALFF in the right precentral gyrus, left superior frontal gyrus (SFG), and orbital frontal cortex (OFC). This decrease was significantly associated with a decrease in SI symptoms following ECT treatment. Both studies enrolled patients with severe baseline depression and severe SI and employed a similar ECT protocol. Participants underwent eight to twelve sessions of ECT, which were dosed using the half age method [51]. This technique computes half of the patients age as a fraction of the total stimulus dose, for instance, delivering 30% of the standard stimulus dose for a 60-year-old patient. Li X and colleagues [36] conducted their study with adolescents, while Wang X and colleagues [47] enrolled young adults. These results merit further investigation of reduced fALFF in the frontal cortex as a potential neural biomarker of SI treatment response following ECT.

Three EEG studies explored the neurophysiological effects of MST [48–50]. Sun and colleagues found a TMS-EEG biomarker of SI treatment response following MST in two separate analyses using overlapping datasets [49, 50]. The study was conducted using an open-label design and recruited patients with TRD and mild-to-moderate SI. One analysis reported a reduction in long interval cortical inhibition (LICI), a measure of cortical inhibition, in the frontal and central electrodes following paired-pulse stimulation over the left dorsolateral prefrontal cortex post-MST treatment. This reduction in LICI was significantly associated with an improvement in SI symptoms following treatment [50]. Similarly, the other analysis found that increased LICI values and higher (more negative) N100 amplitude, another measure of cortical inhibition, at baseline was significantly associated with a stronger improvement in SI symptoms following MST treatment [49]. These findings suggest that MST might reduce SI through inhibitory mechanisms in the frontal cortex. The third study investigating MST response [48] identified four baseline EEG states that significantly predicted an improvement in SI following MST treatment. The EEG microstates encompassed brain networks in the fronto-occipital regions. Although these MST studies employed different neurophysiological approaches compared to the ECT studies, this evidence suggests that both MST and ECT may modulate overlapping brain networks. In sum, these findings provide promising evidence for the involvement of the prefrontal cortex in the anti-suicidal mechanisms of seizure therapies.

### Ketamine

Ketamine is a dissociative anesthetic agent. It is an N-methyl-D-aspartate (NMDA) receptor antagonist and alters excitatory inhibitory balance in the brain by reducing the binding of glutamate, an excitatory neurotransmitter [52]. Ketamine can be administered through various routes including intravenous, intranasal, intramuscular, and oral methods. Intravenous ketamine offers 100% bioavailability, whereas oral ketamine is estimated to have approximately 20% bioavailability, indicating that only 20% of the drug enters the systemic circulation [53]. Nine studies were identified that explored the neurophysiological basis of improvement in SI following ketamine treatment (Table 2). Four studies used resting-state fMRI [31, 33, 39, 54], three used EEG [34, 40, 55], one used PET [38] and one used MEG [41]. Three of the nine studies were randomized controlled trials (RCT), whereas the other six trials were non-randomized open label studies. Three studies administered oral ketamine, with dosages ranging from 0.5 mg/kg to 3.0 mg/kg, and seven studies administered IV-ketamine with a dose of 0.5 mg/kg. Three articles studied a single session of ketamine [38, 39, 41], while the remaining three studies conducted six sessions spread out over two to six weeks [31, 40, 54]. The results of the single infusion studies should be interpreted with caution, as a full treatment course of IV-ketamine is most often described as at least six sessions [56]. Seven out of nine studies found a significant neural biomarker associated with improvement in SI following ketamine treatment.

**Table 2:** Neurophysiological biomarkers of SI improvement following ketamine and rTMS.

Ketamine	Imaging Method	Treatment protocol (1. Type 2. Sessions, 3. Dose	Sample size	Population (1. Diagnosis, 2. Baseline SI, 3. concomitant medication, 4. Age)	SI Response (1. Scale, 2. Pre SI 3. Post SI, 4. *P*-value)	Design	Main Result
Ballard, [38]	PET	1. IV-ketamine 2. Single Infusion 3. 0.5 mg/kg	19	1. TRD 2. Not reported 3. Medication free for ≥ 2 weeks before ketamine infusion 4. 48 ± 12	1. HAMD Item-3 2. Not reported 3. Not reported 4. Not reported	Open label	Decreases in SI were significantly associated with decreased rCMRglu in the infralimbic cortex.
Liu, [54]	fMRI	1. IV-ketamine 2. 6 sessions 4. 0.5 mg/kg	39	1. TRD 2. 9.0 ± 3.5 3. Concurrent medications permitted 4. MDD (36.5 ± 12.1), HC (31.4 ± 8.0)	1. BSSI-5 2. 9.0 ± 3.5 3. 6.0 ± 2.3 4. *p* < 0.001	Open label	Increased baseline resting state functional connectivity between left amygdala and right putamen, right amygdala and right putamen, and left amygdala and right midcingulate cortex were significantly associated with decreases in SI.
Gilbert, [41]	MEG	1. IV-ketamine 2. Single infusion 3. 0.5 mg/kg	29	1. TRD 2. 0.343 (±0.04) 3. Medication free for ≥ 2 weeks before ketamine infusion 4. 35.8 (±10)	1. BDI Item-9; MADRS Item-10 2. 0.343 ± 0.04 3. 0.268 ± 0.04 4. *p* < 0.05	RCT	Changes in SI following treatment are not significantly associated with functional connectivity between anterior insula and ACC
Chen, [31]	fMRI	1. IV-ketamine 2. 6 sessions 3. 0.5 mg/kg	40	1. MDD or BD 2. 5.53 (±2.69) 3. Stable medication dose for ≥ 4 weeks before ketamine infusion 4. 32.85 (±11.36)	1. BSSI 2. 5.53 ± 2.69 3. Not reported 4. Not reported	Open label	Increased functional connectivity between right perigenual ACC and left middle occipital gyrus is associated with an improvement in SI following Ketamine treatment
De la Salle, [40]	EEG	1. IV-ketamine 2. 1 randomized (ketamine vs midazolam) then 6 open label 3. 0.5 mg/kg	24	1. TRD 2. 2.96 (±1.4) 3. Medication free for ≥ 6 weeks before ketamine infusion 4. 41.7 ± 12.3	1. MADRS Item-10 2. 2.96 ± 1.4 3. 0.4 ± 0.9 4. Not reported	RCT	Increased baseline theta band activity in the subgenual ACC and rostral ACC significantly predicts decreased SI 1 day post infusion. Increased baseline alpha band parieto-occipital power predicted a decrease in SI 4 weeks after infusion
Can, [33]	fMRI	1. Oral Ketamine 2. Six 3. 0.5 mg/kg, titrated up between 0.1–0.5 mg/kg	28	1. Chronic SI (BSSI > 6) 2. BSSI mean 37.0 (±2.1) 3. Not reported 4. 43.8 (±14.3)	1. BSSI 2. 19.7 ± 5.1 3. 4.7 ± 7.3 4. Not reported	Open label	The connectivity of the right caudate with 9 regions was positively associated with an improvement in SI following treatment
Can, [34]	EEG	1. Oral Ketamine 2. Six sessions 3. 0.5 mg/kg, dose increase by up to 0.5 mg/kg per session	28	1. Chronic SI (BSSI > 6) 2. Responders 18.1 (+−4.3); Non responders 21.9 (+−5.1) 3. Medication kept stable prior to trial start 4. 44.7 (±13.9)	1. BSSI 2. 18.1 ± 4.3 3. 1.7 ± 3.3 4. Not reported	Open label	Increased auditory evoked power in the left central parietal area alpha band and parietal band predicts a decrease in SI following oral ketamine
Anijärv, [55]	EEG	1. Oral Ketamine 2. Six sessions 3. Flexible dose −0.5–3.0 mg/kg titration	25	1. MDD & Chronic SI 2. 20.0 ± 4.7 3. 92% of participants took concomitant medication 4. 46.41 ± 14.12	1. BSSI 2. Not reported 3. Not reported 4. Not reported	Open label	No significant changes in power spectra were associated with an improvement in SI following treatment
Chen, [39]	fMRI	1. IV-ketamine 2. Single infusion 3. 0.2 mg/kg or 0.5 mg/kg	48	1. TRD 2. 2.63 ± 1.67 (MADRS-10) 3. Medication kept stable prior to trial start 4. 43.4 ± 11.9	1. MADRS-10 2. 2.63 ± 1.67 (0.5 mg/kg), 2.67 ± 1.11 (0.2 mg/kg), 3. 1.69 ± 1.35 (0.5 mg/kg), 0.87 ± 0.74 (0.2 mg/kg), 4. Not reported	RCT	Decreased functional connectivity between left dorsal ACC and right ACC is significantly associated with decreased SI following IV-ketamine (0.5 mg/kg). Increased functional connectivity between right DLPFC and left superior parietal cortex was associated with decreased SI following ketamine, dosed at 0.2 mg/kg.

rTMS	Imaging Method	Treatment protocol (1. Type 2. Sessions, 3. Timeframe, 4. Dose, 5. Target	Sample size	Population (1.Diagnosis, 2. Baseline SI, 3. concomitant medication, 4. Age	SI Response (1. Scale, 2. Pre SI 3. Post SI, 4. P)	Design	Main Result
Baeken [58]	fMRI	1. aiTBS 2. 20 3. Four days (5 sessions/day) 4. 110% of resting MT 5. Left DLPFC	44	1. TRD 2. 13.66 ± 11.53 3. Medication free for ≥ 2 weeks before treatment start 4. 38.73 ± 11.65	1. BSSI 2. 13.66 ± 11.53 3. 6.05 ± 9.69 4. Not reported	RCT	Increased sgACC-mOFC functional connectivity during aiTBS treatment is associated with decreases in hopelessness, but not with changes in SI
Baeken [59]	fMRI	1. aiTBS 2. 20 3. Four days (5 sessions/day) 4. 110% of resting MT 5. Left DLPFC	45	1. MDD 2. 10 ± 16.00 (IQR) 3. Medication free for ≥ 2 weeks before treatment start 4. 44.00 (±19.00)	1. BSSI 2. Median: 6 (IQR: 16.5) 3. Median: 0.00 (IQR: 12.50) 4. *p* = 0.06	RCT	In placebo aiTBS, decreases in SI were accompanied by decreases in perfusion in the bilateral frontal cortices and the superior frontal gyrus
Tang [61]	fMRI	1. iTBS 2. 50 3. 10 sessions daily for 5 consecutive days 4. 90% resting MT 5. Individualized target based on connectivity between DLPFC and ACC	15	1. MDD 2. 14.8 (+−5.73) 3. All patients started venlafaxine or duloxetine at start of study 4. 25.8	1. BSSI 2. 14.8 ± 5.73 3. 2.13 ± 4.77 4. *p* < 0.001	Open label	A decrease in the functional network connectivity between the default mode network and precuneus network is associated with a decrease in SI following treatment
Li [60]	fMRI	1. TMS SAINT protocol 2. 50 3. 10 sessions per day for 5 consecutive days 4. 90% of MT 5. Individualized target based on connectivity between DLPFC and ACC	26	1. MDD 2. 17.63 ¬ ± 7.06 3. All patients started venlafaxine or duloxetine at start of study 4. Not reported	1. BSSI 2. 17.63 ± 7.06 3. 3.39 ± 4.53 4. *p* < 0.001	Open label	A decrease in SI following treatment is significantly correlated with an increase in connectivity between the hippocampus and insula
Barredo [32]	fMRI	1. 5 Hz TMS 2. Up to 40 sessions (mean = 36(±6)) 3. Up to 8 weeks 4. 120% of MT 5. Left DLPFC	25	1. MDD and PTSD 2. Not reported (16 patients reported SI pretreatment) 3. Medication stable for ≥ 6 weeks before treatment start 4. 52.4 ± 10	1. IDS-SR Item-18 2. Not reported 3. Not reported 4. Not reported	Open label	Decreases in SI following treatment is significantly correlated with decreased functional connectivity between the right frontal projecting striatum and the right frontal pole

*PET* positron emission tomography, *fMRI* functional magnetic resonance imaging, *MEG* magnetoencephalography, *EEG* electroencephalogram, *IV* intravenous, *TRD* treatment resistant depression, *MDD* major depressive disorder, *HC* healthy control subjects, *BP* bipolar disorder, *BSSI* beck scale for suicidal ideation, *HAMD* hamilton depression scale, *BDI* Beck’s depression inventory, *MADRS* montgomery–asberg depression rating scale, *RCT* randomized controlled trial, *rCMRglu* regional cerebral glucose metabolic rates, *ACC* anterior cingulate cortex, *DLPFC* dorsolateral prefrontal cortex, *rTMS* repetitive transcranial magnetic stimulation, *aiTBS* accelerated intermittent theta burst stimulation, *MT* motor threshold, *IQR* inter quartile range, *PTSD* post-traumatic stress disorder, *IDS-SR* inventory for depressive symptomatology, *OFC* orbitofrontal cortex.

Most studies (six out of nine) investigated the association between the cingulate cortex and improvements in SI following ketamine treatment. Four out of the six studies found a significant neural biomarker of SI improvement in the cingulate cortex of which three were specifically in the anterior cingulate cortex. Interestingly, none of the studies that used oral ketamine found a neural biomarker of SI improvement in the cingulate cortex, which is in strong contrast to the four (out of 5) studies using IV-ketamine. Chen [39] found a positive correlation between the improvement in SI and the increase in functional connectivity of the left dorsal anterior cingulate cortex (ACC) and right ACC, following a single infusion of IV-ketamine. Although this was a well-powered (*n* = 48) RCT, the study only investigated a single infusion at different doses (0.5 mg/kg or 0.2 mg/kg). Chen and colleagues [31] conducted an open-label trial on 40 patients diagnosed with either unipolar or bipolar depression undergoing six IV-ketamine infusions within two weeks. They found an increase in the functional connectivity between the perigenual ACC and left middle occipital gyrus following the treatment course, which was significantly associated with an improvement in SI symptoms. Using EEG, De La Salle and colleagues [40] found that increased power in the theta band of the subgenual ACC (sgACC) and rostral ACC (rACC) predicted improvement in SI following treatment. In this study, 24 patients with severe depression (MADRS > 25) were randomized to one infusion of either IV-ketamine or midazolam, and subsequently received six doses of open label IV-ketamine. Gilbert JR [41] studied insula-ACC connectivity using magnetoencephalography (MEG) in 29 individuals randomized to a single infusion of 0.5 mg/kg of IV-ketamine. They did not find a significant association between the insula-ACC functional connectivity and the change in SI following treatment.

In summary, activity and connectivity of the ACC shows initial promise as a potential neurophysiological biomarker of SI treatment response following IV-ketamine. Although Gilbert and colleagues found no significant correlation between insula ACC connectivity and SI treatment response following IV-ketamine, the finding should be interpreted with caution as patients did not undergo a full treatment course. Additionally, none of the studies using oral ketamine found a significant neural biomarker of SI treatment response in the cingulate cortex. This is an important finding that might highlight the distinct treatment mechanisms of intravenous and oral ketamine.

### Repetitive transcranial magnetic stimulation

Repetitive Transcranial Magnetic Stimulation (rTMS) is a well-tolerated non-invasive neuromodulation technique utilizing electromagnetic stimulation to modulate neural activity in the cortical regions of the brain in awake patients. It is FDA approved for the treatment of major depressive disorder, obsessive compulsive disorder, smoking cessation and migraines [57]. rTMS is an overarching term for a family of interventions that vary in terms of coil shape, and the number, frequency, intensity, and continuity of pulses and frequency of treatment sessions. This review highlights two main protocols: 5 Hz rTMS and accelerated intermittent theta burst stimulation (aiTBS). 5 Hz rTMS is a low frequency stimulation protocol usually delivered as one daily session, lasting around 30 min, for eight weeks [32]. The aiTBS protocol is a more intensive form of rTMS in which patients undergo five to ten daily sessions for a total of five days and receive bursts of pulses delivered at a theta frequency (4–7 Hz).

Of the five studies that met the inclusion criteria for this review (Table 2), four used an aiTBS protocol targeting the left dorsolateral prefrontal cortex [58–61] and one study used a 5 Hz rTMS protocol [32]. Three of the five studies found a significant neural biomarker for improvement in SI following rTMS treatment [32, 60, 61]. One study found a significant neural biomarker associated with the placebo response, meaning an improvement in SI symptoms following a sham intervention [59]. Another study found a significant neural biomarker that was associated with the change in hopelessness following rTMS treatment, but did not find a neural biomarker associated with SI [58]. Hopelessness is a prominent component of SI and was quantified using the Beck Hopelessness Scale (BHS) [62] by Baeken and colleagues [58]. All studies used resting state fMRI to study neurophysiological biomarkers, of which two studies used a seed-based functional connectivity approach [32, 58], with seeds in the sgACC and striatum/thalamus, respectively.

There is prominent heterogeneity in the neural biomarkers identified between the studies, which may be attributed to differences in TMS protocols, including variability in TMS pulse intensity (90–120% of motor threshold), frequency (5 Hz vs 50 Hz), and number of treatment sessions (20–50 sessions). Additionally, studies varied in terms of inclusion of comorbid disorders, definition of regions of interest and concomitant medications. Two studies [60, 61] investigated the effects of an aiTBS protocol in combination with a concurrent SNRI medication (either venlafaxine or duloxetine). Tang and colleagues [61] found an increase in functional connectivity between the precuneus network and the default mode network that was significantly correlated with an improvement in SI symptoms following the iTBS treatment. Li and colleagues (2024) found a significant increase in connectivity between the hippocampus and insula associated with a decrease in SI following an accelerated TMS protocol. These findings should be interpreted with caution as all patients received either venlafaxine or duloxetine at the beginning of the treatment course. This complicates the interpretation of the results as the neurobiological changes could be attributed to the pharmacological intervention or TMS.

Baeken and colleagues [58] conducted a RCT on 44 patients receiving aiTBS or sham stimulation targeting the left DLPFC. The patients receiving active stimulation did not yield a significant association between the improvement in SI and the functional connectivity in the sgACC. However, increased functional connectivity between the sgACC and the medial orbitofrontal cortex was significantly associated with a decrease in hopelessness, measured by the BHS. Using the same dataset, Baeken and colleagues [59] conducted a secondary analysis on placebo response following accelerated iTBS. They found that reduction in SI following sham stimulation was significantly associated with a decrease in perfusion in the bilateral frontal cortices and the SFG. These results may highlight physiological correlates of improvement in SI that are independent from the treatment mechanisms.

Barredo and colleagues [32] studied 25 MDD patients with comorbid PTSD undergoing 5 Hz TMS treatment for up to eight weeks. They found a decrease in functional connectivity between the right frontal projecting striatum seed and the right frontal pole, which was significantly associated with a decrease in SI symptoms. The results of this study should be considered in the context of patients with comorbid PTSD diagnosis and the utilization of a single item on the IDS-SR to quantify SI severity. Additionally, the sample consisted of patients with mostly mild SI, including 10 patients with mild SI, five with moderate SI, and two with severe SI.

### Psychopharmacological interventions

Three studies used EEG to identify neurophysiological biomarkers associated with a change in SI following antidepressant treatment [35, 42, 43] (Table 3). The results demonstrate no clear overlapping findings, which may be attributed to vastly distinct methodological approaches between the studies, including different antidepressant medications, neurophysiological methods, follow-up timepoints, regions of interest and SI assessments used. Hunter and colleagues [42] conducted a RCT with 72 individuals that received either fluoxetine (20 mg), venlafaxine (150 mg) or placebo. 48 h after treatment initiation they found that there was a decrease in midline-and-right-frontal (MRF) cordance, which was significantly associated with worsening SI. Cordance represents a quantitative EEG (QEEG) measure that combines both absolute and relative EEG power, thereby enhancing the signal-to-noise ratio in comparison to absolute EEG measures. Lewis and colleagues [35] studied 10 adolescents that were either taking fluoxetine (eight), escitalopram (one) or bupropion (one). TMS-EEG was administered at baseline and after eight weeks post-treatment to study changes in neurophysiology. The results show that the increase in cortical inhibition (LICI) post-treatment significantly correlated with an improvement in SI following 8 weeks of antidepressant treatment. Iosifescu and colleagues [43] studied 82 MDD patients with mild SI taking an SSRI. 53 patients took escitalopram, seven fluoxetine, seven paroxetine, five citalopram, five sertraline, and five venlafaxine. The authors found that frontal theta and alpha asymmetry was significantly higher post treatment in patients that experienced worsening SI.

**Table 3:** Neurophysiological biomarkers of SI improvement following psychotherapy and antidepressants.

Psychotherapy	Imaging Method	Treatment protocol (1. Type 2. Details	Sample	Population (1.Diagnosis, 2. Baseline SI, 3. concomitant medication, 4. Age	SI Response (1. Scale, 2. Pre SI 3. Post SI, 4. P)	Design	Main Result
Shu, [63]	fMRI	1. CBT with fluoxetine or just fluoxetine 2. CBT once per week for 8 weeks	40	1. MDD 2. CBT (43.48 ± 10.66), MG (42.11 ± 7.17) 3. No treatment within 1 month of study 4. 23.63 ± 3.64 (MG), 22.24 ± 2.95 (CBT)	1. BSSI 2. 43.48 ± 10.66 3. 5.95 ± 5.25 4. *p* = 0.005	RCT	Increases in fALFF values in the medial prefrontal cortex were significantly associated with a decrease in SI following 8 weeks of CBT treatment
Shu, [64]	fMRI	1. CBT with fluoxetine or just fluoxetine 2. CBT once per week for 8 weeks	84	1. MDD 2. CBT (43.2 ± 9.8), MG (42.5 ± 7.2) 3. No treatment within 1 month of study 4. CBT (22.1 ± 2.8), MG (23.4 ± 3.6), HC (23 + −2.2)	1. BSSI 2. 43.2 ± 9.8 3. 8.5 ± 8.6 4. *p* = 0.023	RCT	Increased functional connectivity between the right precuneus and the right superior frontal cortex was significantly associated with a decrease in SI following 8 weeks of CBT treatment.

Antidepressants	Imaging Method	Treatment protocol (1. Type 2. Duration, 3. Dose)	Sample size	Population (1. Diagnosis, 2. Baseline SI, 3. concomitant medication, 4. Age	SI Response (1. Scale, 2. Pre SI 3. Post SI, 4. P)	Design	Main Result
Hunter, [42]	EEG	1. Fluoxetine, venlafaxine, or Placebo 2. 8 weeks with 1 week placebo lead-in 3. Fluoxetine −20 mg, venlafaxine −150 mg	72	1. MDD 2. Not reported 3. No psychotropic medication for 2 weeks prior to study entry 4. 41.7 ± 12.1	1. HAMD-17 Item-3 2. Not reported 3. Not reported 4. Not reported	RCT	Decreased MRF cordance at 48 h post treatment initiation is significantly associated with worsening SI
Lewis, [35]	TMS- EEG	1. Antidepressants (8 taking fluoxetine; 1 escitalopram; 1 bupropion) 2. 2–20 weeks follow up (mean:8) 3. Not reported	10	1. MDD 2. 7.00 ± 6.88 3. Not reported 4. 15.5 ± 1.18	1. C-SSRS 2. 7.00 ± 6.88 3. 3.10 ± 4.43 4. Not reported	Open label	Increased cortical inhibition (LICI-100) is significantly associated with an improvement in SI following treatment
Iosifescu, [43]	EEG	1. SSRI (65% took escitalopram) 3. 8 weeks 4. Variable dosages	82	1. MDD 2. 0.8 ± 0.8 3. No psychotropic medications for 1 week prior to trial 4. 35.9 ± 13.0	1. HAM-D Item-3 2. 0.8 ± 0.8 3. 0.5 ± 0.7 4. Not reported	Open label	Frontal theta and alpha asymmetry is significantly higher post treatment in patients that experienced worsening SI

*fMRI* functional magnetic resonance imaging, *CBT* cognitive behavioral therapy, *MDD* major depressive disorder, *BSSI* beck scale for suicidal ideation, *MG* medication group, *RCT* randomized controlled trial, *fALFF* fractional amplitude of low-frequency fluctuations, *TMS* repetitive transcranial magnetic stimulation, *EEG* electroencephalogram, *TMS-EEG* combined TMS and EEG, *SSRI* selective serotonin reuptake inhibitor, *MDD* major depressive disorder, *HAMD* hamilton depression scale, *C-SSRS* Columbia suicide severity scale, *MRF* cordance midline-and-right-frontal (MRF) cordance, *LICI* long interval cortical inhibition.

In summary, although all three studies found a significant neural biomarker of change in SI following treatment, two out of the three neural biomarkers were associated with worsening SI post-treatment. These findings could provide insight into the neural mechanisms of the adverse effects of antidepressants.

### Psychotherapy

Two articles explored fMRI biomarkers of improvement in SI following psychotherapy [63, 64] (Table 3). The studies used overlapping datasets but employed distinct methodological approaches. The first analysis revealed a significant association between increased fALFF values in the medial prefrontal cortex and a decrease in SI following eight weeks of CBT treatment. The second analysis reported an increase in functional connectivity between the right precuneus and the right superior frontal cortex that was significantly associated with a decrease in SI following eight weeks of CBT treatment. These results offer an initial indication for the potential involvement of the prefrontal cortex in the beneficial effects of psychotherapy for SI.

## Discussion

This systematic review summarizes the results of 24 articles on the neurophysiological basis of treatment-induced changes in SI. We found convergence in the involvement of the anterior cingulate cortex in the anti-suicidal effects of IV-ketamine, and in the prefrontal cortex in the anti-suicidal effect of seizure therapies. These findings provide valuable insights into the neurophysiological basis of SI in patients with MDD and may guide the optimization of treatments targeting SI in future clinical trials. However, the included studies demonstrate substantial heterogeneity in methodological approaches used, limiting the synthesis of results across studies. Therefore, more large-scale studies with standardized methodological approaches are needed to replicate the findings of this review.

The majority of articles included in this review were published in the past five years, reflecting the growing interest in the neurophysiological basis of SI. This also highlights the relevance of this review for informing the methodology and research questions of future studies. Also, only two of the 24 studies focused their analyses on adolescents, a population disproportionately affected by increasing suicide rates [65, 66], indicating the urgent need for more research on the neurophysiology of SI in adolescents.

**Fig. 3:**
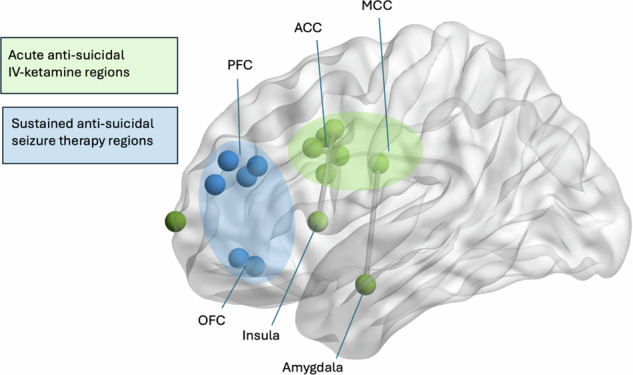
Conceptual figure illustrating the neurophysiological basis of the anti-suicidal treatment effects of IV-ketamine (in green) and seizure therapies (in blue). PFC prefrontal cortex, ACC anterior cingulate cortex, MCC medial cingulate cortex, OFC orbitofrontal cortex.

The results of this review point towards the potential involvement of the anterior cingulate cortex in the anti-suicidal effects of IV ketamine, with four independent studies, using multiple different neurophysiological approaches, identifying a significant neural biomarker in the cingulate cortex (Fig. 3). This finding complements prior research showing altered functional connectivity in the cingulate cortex in patients with SI [67]. Moreover, prior research has demonstrated the inhibitory effects of ketamine in the ACC [68], as well as an association between ketamine’s anti-depressant properties and activity in the subgenual and dorsal regions of the ACC [69]. The involvement of the ACC in affect regulation is well-characterized [70]. Notably, this review revealed that involvement of the ACC in the anti-suicidal effect of ketamine was limited to IV-ketamine and was not evident in three studies utilizing oral ketamine. While there is no prior research comparing the neurophysiological mechanisms of IV-ketamine with oral ketamine, existing literature suggests a more robust anti-suicidal effect of IV-ketamine compared to oral ketamine [71]. These differential clinical effects may be attributed to the association of the ACC with emotional dysregulation and mental pain [72, 73], concepts which strongly drive symptoms of SI. Accordingly, this superior effect of IV-ketamine may be linked to the mechanistic engagement of the ACC specific to IV administration. However, the results of this review should be interpreted with caution as the studies used distinct methodologies and explored different regions of interest. Further research is needed to compare the neurophysiological mechanisms of oral compared to IV administration of ketamine.

The findings of this review suggest a potential involvement of the frontal cortex in the anti-suicidal effect of ECT and MST (Fig. 3). All studies (five) that explored the treatment effects of ECT and MST found a significant neural biomarker in the frontal cortex (four in the prefrontal cortex) that was associated with an improvement of SI post-treatment. This is in line with previous research that has shown modulation of activity in the frontal cortex as the main mechanism of the anti-depressant effects of ECT [74]. The converging results between ECT and MST presented in this review suggest overlapping neural mechanisms between these two treatment approaches for the treatment of SI. Further research is needed to identify the neural basis of the differential side effect profile of ECT and MST, as there is evidence that patients experience fewer side effects as a result of MST compared to ECT [46].

The rTMS literature relevant to this review showed no convergence among the neurophysiological results. This could be attributed to the heterogeneity across methodologies used, including the rTMS protocol, concomitant medication, and regions of interest. Increased connectivity between the insula and hippocampus was identified as a neural biomarker of an improvement in SI following treatment by Li and colleagues [60]. This findings is consistent with a previous review by Schmaal and colleagues [30], which highlighted the insula and hippocampus as critical regions in the neurophysiological basis of SI. The authors argued that the insula’s role in interoceptive awareness and mental pain, along with the hippocampus’s role in stress response and memory biases, contributes to SI [30]. Baeken and colleagues [58] found a significant association of increased connectivity between ACC and medial OFC connectivity and an improvement in hopelessness, but not in SI, following TMS. The involvement of the medial OFC in reward processing might explain its specific association with hopelessness, an emotion closely linked to dysfunctional reward processing [75]. Future studies should aim to replicate the results of current studies using more controlled designs that can isolate the effects of rTMS. Baeken and colleagues [59] identified a neural biomarker associated with placebo treatment response following sham aiTBS. Consistent with previous studies that highlight a higher placebo response for SI compared to other mood disorder symptoms [76], this finding underscores the importance of sham controlled trials to effectively control for neurophysiological placebo effects in SI research. Further research on the neurophysiological correlates of the SI placebo response is needed to characterize the placebo response across treatment modalities.

There were insufficient studies utilizing antidepressants to study the neurophysiological basis of an improvement in SI following treatment. The studies were methodologically distinct as there were different antidepressants utilized, and distinct neurophysiological approaches. Hunter and colleagues conducted a methodologically rigorous study which found a neurophysiological biomarker at 48 h post treatment initiation associated with symptom worsening. Upon further replication this could be a clinically useful early indicator of worsening SI following treatment. Future studies should compare and contrast the neurophysiological differences between treatment-induced worsening of SI and SI present at baseline in suicidal individuals.

The findings of this review must be considered in light of several limitations. Reviews of this nature are inevitably confounded by publication bias, leading to an underrepresentation of studies that do not identify a significant neural biomarker of treatment response. Also, fifteen of the studies included in this review were uncontrolled trials. It is important to note the limited ability of uncontrolled trials to draw casual inferences, which is underscored by Baeken and colleagues [59], who found a significant neural biomarker associated with placebo response. Additionally, most of the studies were of exploratory nature, lacked a clearly defined a priori hypothesis, and were limited to small sample sizes. Further large-scale, placebo-controlled trials with a priori hypotheses are needed to identify robust neural biomarkers of SI. The assessments used to quantify SI are a further limitation of several studies included in this review. Many studies used only a single SI item on a depression questionnaire to quantify SI severity. Although Chung and colleagues [77] demonstrated moderately strong associations of the SI item on the PHQ-9 with comprehensive SI scales, the limited variance of the single-item scales impacts the reliability of correlation analyses. Overall, the comparison of results was challenging due to prominent heterogeneity in methodological approaches employed for the neurophysiological assessments and treatment protocols. For example, several studies investigated regions of interest that did not overlap across studies, and not all studies delivered full treatment courses (e.g. single IV-ketamine infusions). Despite these limitations, the findings of this review suggest preliminary treatment-specific evidence for the neurophysiological biomarkers associated with an improvement in SI following ECT and IV-ketamine.

In summary, this review suggests the involvement of the ACC in the anti-suicidal effect of IV-ketamine and an association between the PFC and the therapeutic response of convulsive therapies in patients diagnosed with MDD. These potentially distinct neurobiological mechanisms may explain in part the differences in the clinical effects of IV-ketamine and ECT. IV-ketamine often elicits an immediate anti-suicidal effect that does not sustain longer than a few days [15]. In contrast, ECT has a more gradual yet persistent anti-suicidal effect, with most patients remitting after two to three weeks and sustaining remission over months or years [12]. The temporally distinct effects of ECT and IV-ketamine could be attributed to the differential functionalities of the ACC and PFC. The ACC is linked to pain perception [73] and emotional processing [72], potentially underpinning IV-ketamine’s acute effects on mental pain [78] and emotional dysregulation [79]. Conversely, the PFC is associated with inhibition and executive functioning, encompassing memory, planning, attention, and cognitive flexibility. ECT’s persistent anti-suicidal effects may stem from its modulation of the PFC, addressing factors such as cognitive bias [80] and mental inflexibility [81]. Although these theories require rigorous testing in a controlled clinical trial, our initial insights suggest that the PFC and ACC both play unique roles in the treatment mechanisms for the alleviation of SI.

The findings of this review offer several important implications for future research on SI related biomarkers and the treatment of SI: a) Researchers should attempt to standardize methodological approaches to facilitate the synthesis of results, which is essential for validating robust biomarkers across datasets. b) Researchers should focus on the prefrontal cortex and anterior cingulate cortex when designing interventions to treat SI that are informed by neurophysiological evidence. c) Future studies should test whether the findings of this review are specific to SI in MDD, or if they could be considered transdiagnostic, seeing that most studies in this review (92%) required MDD diagnosis as an inclusion criterion.

## Conclusion

This systematic review summarizes the current literature on the neurophysiological basis of changes in SI following treatment. Although this topic has gained attention in the past 5 years, there is limited convergence of findings. Study methodologies vary, and many studies are limited by simultaneous interventions, non-randomized study design, small sample sizes, incomplete treatment protocols and unvalidated SI assessments. The results of this systematic review suggest a possible involvement of the prefrontal cortex in the anti-suicidal effect of seizure therapies. Furthermore, the anterior cingulate cortex might be implicated in the anti-suicidal effects of IV-ketamine. Notably, there is no convergence in the findings between oral and intravenous ketamine, indicating possibly distinct neurophysiological mechanisms between these methods of administration. Future studies should involve large randomized trials and may seek to employ next generation tests of neuroplasticity underlying the therapeutic effects of SI treatments [82]. Ultimately, validated neurophysiological predictors of an improved treatment outcome could inform clinical decisions on the best suited treatment for a patient with a given neurophysiological profile. Moreover, these results may inform the development of novel treatments that target SI with greater precision, while optimizing maintenance of treatment response, and minimizing side effects.

## Supplementary information

Supplemental Materials
