# Supplementary material for: Neurophysiological biomarkers of treatment response in suicidal ideation: a systematic review
Source: Transl Psychiatry. 2025 Nov 17;15:473. doi: 10.1038/s41398-025-03477-2 (PMC12623415; doi:10.1038/s41398-025-03477-2)
Supplement: Supplementary file 1 — Supplemental Materials [file 41398_2025_3477_MOESM1_ESM.docx]

**Supplemental Materials 1**

Pubmed search statement:

("Suicidal Ideation"[Mesh] OR suicidal ideation [TIAB] OR suicidal thoughts [TIAB] OR suicidality [TIAB] OR “suicide, attempted”[Mesh] OR “attempted suicide”[TIAB] OR “suicide attempt”[TIAB] OR “suicide attempts”[TIAB] OR “suicidal behavior”[TIAB])

AND (“neurophysiology”[Mesh] OR neurophysiology [TIAB] OR "functional neuroimaging" [Mesh] OR functional neuroimaging [TIAB] OR electroencephalogram [TIAB] OR electroencephalography [TIAB] OR “electroencephalography”[Mesh] OR EEG [TIAB] OR transcranial magnetic stimulation electroencephalography [TIAB] OR "TMS-EEG" [TIAB] OR "TMS EEG" [TIAB] OR "functional magnetic resonance imaging" [TIAB] OR “fMRI” [TIAB] OR "Positron-Emission Tomography"[Mesh] OR "Positron emission tomography" [TIAB] OR "positron-emission tomography" [TIAB] OR PET [TIAB] OR "intracranial electroencephalogram" [TIAB] OR iEEG [TIAB] OR "tomography, emission-computed, single-photon" [MESH] OR "single-photon emission computed tomography" [TIAB] OR SPECT [TIAB] OR “functional near-infrared spectroscopy” [TIAB] OR "TMS-EMG" [TIAB] OR "TMS EMG" [TIAB] OR "Magnetoencephalography"[Mesh] OR “Magnetoencephalography” [TIAB] OR “MEG” [TIAB])

AND (“transcranial magnetic stimulation” [MESH] OR “transcranial magnetic stimulation” [TIAB] OR “TMS” [TIAB] OR rTMS [TIAB] OR “repetitive transcranial magnetic stimulation” [TIAB] OR “TBS” [TIAB] OR “theta burst stimulation” [TIAB] OR “aTBS” [TIAB] OR “accelerated theta burst stimulation” [TIAB] OR "magnetic field therapy"[MeSH Terms] OR “magnetic seizure therapy” [TIAB] OR MST [TIAB] OR “electroconvulsive therapy” [TIAB] OR “ECT” [TIAB] OR “deep brain stimulation” [TIAB] OR DBS [TIAB] OR “vagal nerve stimulation” [TIAB] OR VNS [TIAB] OR “ketamine” [MeSH Terms] OR ketamine [TIAB] OR esketamine [TIAB] OR “intravenous ketamine” [TIAB] OR “IV-Ketamine” [TIAB] OR “transcranial direct-current stimulation” [TIAB] OR tDCS [TIAB] OR "Psychotherapy"[Mesh] OR "Cognitive Behavioral Therapy"[Mesh] OR psychotherapy [TIAB] OR “cognitive–behavioral therapy” [TIAB] OR “antidepressants” [TIAB] OR "Dose-Response Relationship, Drug"[Mesh] OR “antidepressive agents”[Mesh] OR "Anticonvulsants"[Mesh] OR "Selective Serotonin Reuptake Inhibitors"[Mesh] OR "Antipsychotic Agents"[Mesh] OR "Hypnotics and Sedatives"[Mesh] OR "Anti-Anxiety Agents"[Mesh] OR “Selective serotonin reuptake inhibitors” [TIAB] OR “SSRI” [TIAB] OR antipsychotics [TIAB] OR “atypical antipsychotics”[TIAB] OR anticonvulsants [TIAB] OR “mood stabilizers” [TIAB] OR sedatives [TIAB] OR anxiolytics [TIAB] OR hypnotics [TIAB] OR "treatment outcome"[Mesh])
